# Remarks on phylogeny and molecular variations of criconematid species (Nematoda: Criconematidae) with case studies from Vietnam

**DOI:** 10.1038/s41598-022-18004-2

**Published:** 2022-09-01

**Authors:** Huu Tien Nguyen, Thi Duyen Nguyen, Thi Mai Linh Le, Quang Phap Trinh, Wim Bert

**Affiliations:** 1 Institute of Ecology and Biological Resources, Vietnam Academy of Sciences and Technology, 18 Hoang Quoc Viet, Cau Giay, 100000 Hanoi, Vietnam; 2 Graduate University of Science and Technology, Vietnam Academy of Sciences and Technology, 18 Hoang Quoc Viet, Cau Giay, 100000 Hanoi, Vietnam; 3 Nematology Research Unit, Department of Biology, Ghent University, K.L, Ledeganckstraat 35, 9000 Ghent, Belgium

**Keywords:** Genetic variation, Evolution

## Abstract

The family Criconematidae is a remarkable group of nematodes, containing roughly 600 nominal root-ectoparasitic species, of which many species are known to be significant agricultural pests. Strikingly, our phylogenetic analyses based on 18S, D2-D3 of 28S rRNA, and *COI* mtDNA sequences of criconematid species, supported by tree topology tests (SH and AU tests), revealed that almost all studied genera, including *Criconema*, *Ogma*, *Crossonema*, *Discocriconema*, *Hemicriconemoides*, *Criconemoides*, *Mesocriconema*, and *Lobocriconema*, are not monophyletic groups, a finding that is partly contrary to those of previous studies on these groups. Our results suggest that key morphological characters used in the classification of Criconematidae are the consequence of convergent evolution. It is clear from our studies that the species status of at least 40 sequences of criconematid species from GenBank must be either revised or reconsidered, with analyses based on a polyphasic approach that includes different tree- and distance-based molecular species-delimitation methods (bPTP, GMYC, ABGD1, and ABGD2). Our studies found the ABGD2 output of the automatic barcode method to agree remarkably well with established species delimitations, while in general, the four species-delimitation results corresponding to three barcode regions forwarded significantly more putative species compared to those originally considered. This study also characterised for the first time the populations of *Criconemoides myungsugae* and *Discocriconemella hensungica* associated with Vietnamese ginseng, one of the most precious and rare ginseng varieties in the world. Although these populations are morphologically in agreement with the original descriptions of *C. myungsugae* and *D. hengsungica*, their molecular data display notable variations compared to the sequences deposited in GenBank. These species demonstrate clearly the immense molecular variations that can be observed in several species of the family Criconematidae.

## Introduction

The family Criconematidae, known as ring nematodes, consists of a vast number of nominal species (approximately 600 species in total) that are root-ectoparasitic nematodes^1^. During recent decades, the taxonomy of species belonging to the family Criconematidae has been revised independently by many nematologists, creating conflicting definitions of genera and in certain cases species has been transferred to five or more genera^1–3^. As an example, Taylor^4^ proposed the genus *Criconemoides* based on the type species *Criconemoides morgensis* Hofmaenner et al.^5^. However, De Grisse and Loof considered it as *genus inquirendum* in 1965 and re-established this genus after two years^6,7^. Luc^8^ considered 7 genera, including *Macroposthonia*, *Criconemoides*, *Nothocriconema*, *Lobocriconema*, *Discocriconemella*, *Criconemella*, and *Xenocriconemella*, as synonymous and retained for them the name of *Criconemoides*. However, Luc et al.^2^ stated that the 5 genera, including *Macroposthonia*, *Criconemoides*, *Criconemella*, and *Xenocriconemella*, should be attributed to a single genus (*Criconemella*) and considered the division of Luc^8^ as being too drastic. Siddiqi^9^ and Geraert^1^ did not agree with such a drastic revision and divided the family Criconematidae into 44 and 18 genera, respectively. In this study, we have used the generic name *Criconemoides*, *Discocriconemella*, and other generic names following the classification of Geraert^1^ and Decraemer et al.^3^, given that these represent the most recently-published and widely-accepted opinions on this nematode group.

Since the use of molecular data in taxonomy became possible, several efforts have been made to construct the molecular phylogeny of species in the family Criconematidae, such as studies of Subbotin et al.^10^ based on D2-D3 of 28S rRNA sequences and Powers et al.^11^ based on 18S rRNA sequences. However, molecular data on GenBank are currently only available for a small fraction of the 600 nominal species of Criconematidae, although GenBank is being updated on a regular basis. Besides, Vietnamese ginseng, *Panax vietnamensis*, is an endemic species in Vietnam with very high content of saponins and is considered among the top precious ginseng varieties of the world^12^. This host plant is also considered as a threatened species, meaning studies on pests associated with this plant are of crucial importance in creating a basis for pest management. Our study aimed to: (1) characterise *C. myungsugae* and *D. hengsungica* associated with Vietnamese ginseng based on morphology and molecular data; (2) reconstruct an updated Criconematidae phylogeny using D2-D3 of 28S, 18S rRNA gene, and *COI* gene of mtDNA; (3) reassign unidentified and/or incorrectly classified GenBank sequences to the appropriate species using tree- and distance-based molecular species-delimitation methods; (4) reassess the generic relationships in the Criconematidae using phylogenetic analyses and tree topology tests.

## Materials and methods

Soil samples were collected from the rhizosphere of Vietnamese ginseng (*Panax vietnamensis* Ha & Grushv) in the Western Highlands, Vietnam. Nematodes were extracted using the modified Baerman tray method^13^. For morphological characterisation, nematodes were fixed in TAF for a week and transferred to glycerine to make permanent slides following the method of Seinhorst^14^. Measurements and pictures were taken from nematodes in permanent slides using a Carl Zeiss Axio Lab.A1 light microscope equipped with an Axiocam ERc5s digital camera.

For molecular analyses, a single individual of living nematodes was used for DNA extraction. Living nematodes were cut into several pieces and transferred to a PCR tube with 20 μl of WLB (50 mM KCl;10 mM Tris pH 8.3; 2.5 mM MgCl2; 0.45% NP-40 (Tergitol Sigma); 0.45% Tween-20). Subsequently, the sample was incubated at − 20 °C for 10 min, followed by adding 1 μl proteinase K (1.2 mg ml − 1), incubating in a PCR machine for 1 h at 65 °C and 10 min at 95 °C.

Each polymerase chain reaction (PCR) contained 25 μl Hot Start PCR Master Mix (Promega, Madison, Wisconsin, USA), 1 μl of each forward and reverse primer (10 μM), and 5 μl extracted DNA. 18S rRNA, D2-D3 of 28S rRNA, and *COI* mtDNA regions was amplified using MN18F/Nem_18S_R (5’- CGCGAATRGCTCATTACAACAGC-3’/5’-GGGCGGTATCTGATCGCC-3’), D2A/D3B (5'-ACAAGTACCGTGGGGAAAGTTG-3' / 5'-TCGGAAGGAACCAGCTACTA-3') and JB3/JB4 (5'-TTTTTTGGGCATCCTGAGGTTTAT-3' / 5'-TAAAGAAAGAACATAATGAAAATG-3') primers^11,15,16^. The thermal profiles for amplifying 18S and D2-D3 region was one cycle of 94 °C for 4 min, followed by five cycles of 94 °C for 30 s, 56 °C for 30 s, 72 °C for 2 min, and 45 cycles of 94 °C for 30 s, 54 °C for 30 s, 72 °C for 1 min and finished at 10 °C for 10 min. For *COI* region, the annealing temperature was set to 45°C^17^. Wizard SV Gel and PCR Clean-Up System (Promega, Madison, Wisconsin, USA) were used to purify successful PCR, and sequencing was done commercially by 1st BASE (Asian). The obtained sequences were assembled using Geneious R11 (www.geneious.com). Closely related sequences of species in the family Criconematidae were obtained using BLAST from GenBank^18^. All obtained sequences were aligned using MUSCLE in Geneious R11. Bayesian phylogenetic analysis was executed using MrBayes 3.2.6 add-in in Geneious R11 following Nguyen et al.^19^. HKY + G + I, HKY + I, and GTR + G + I models were selected for constructing respectively 18S, 28S, and COI phylogenetic trees using ModelTeller, a machine learning tool for phylogenetic model selection^20^. Sequences of *Hemicycliophora* (accession number: MG701279, Ạ966471, MW001621, MN628433, MW000897, MG019904) were chosen as outgroups. Obtained phylogenetic trees were viewed and edited using Figtree v1.4.4.

Molecular species-delimitation analyses were performed using three methods including the Bayesian implementation of the Poisson tree processes (bPTP)^21^, generalized mixed-yule coalescent (GMYC)^22^, and Automatic Barcode Gap Discovery (ABGD)^23^. For bPTP and GMYC methods, ultrametric trees were created using BEAST v 1.10.4^24^ with default setting (1 × 10^7^ generations with subsampling every 1 × 10^3^ generations). TreeAnnoator 1.10.4 was used to create the final trees by removing 2000 samples (20%) as burn-ins. The final trees were uploaded to an online server (https://species.h-its.org/) to obtain species-delimitation results following the method of Decraemer et al.^25^. For the ABGD method, aligned sequences in fasta files were uploaded to another online server (https://bioinfo.mnhn.fr/abi/public/abgd/abgdweb.html/) and two best results with smallest prior maximal distances were selected for each dataset (named as ABGD1 and ABGD2).

Monophyly of studied genera was tested using the Shimodaira–Hasegawa (1989) and the approximately unbiased tests (SH and AU tests) in IQ-TREE multicore version 2.2.0^26^. Constrained tree for each genus was created using Notepad +  + v7.5.6 and the same alignments as above were used for the tests.

## Results

### Characterisation of Criconemoides myungsugae (Choi & Geraert, 1975) Loof & De Grisse, 1989 from Vietnam

#### Measurements

All measurements of *Criconemoides myungsugae* from Vietnam are provided in Table 1.

**Table 1:** Morphometrics of *Criconemoides myungsugae* from Vietnam and the world.

Character	*Criconemoides myungsugae* Choi & Geraert^27^				*Criconemoides morgensis* (Hofmanner & Menzel, 1914) Taylor, 1936	*Criconemoides annulatus* Cobb in Taylor, 1936
Source and locality	This study Vietnam	Type population re-described by Choi et al.^28^ South Korea	Eskandari et al., 2010 Iran	Maria et al.^29^ China	Brzeski et al., 2002 Switzerland	Raski & Golden, 1966 United State
n	10	13	18	15	–	9
L	430 ± 34 (395–468)	450 (410–490)	546 (455–630)	527 ± 35.5 (459–574)	510–700	530 (500–550)
a	11.3 ± 0.5(10.6–11.8)	10.9 (10.1–11.4)	11 (9.4–13.3)	12.0 ± 1.3 (9.7–14.5)	–	13 (12–15)
b	4.1 ± 0.2(4.0–4.4)	3.7 (3.6–4.0)	4.4 (3.8–4.8)	4.6 ± 0.3 (4.2–5.1)	–	3.7 (3.4–4.0)
c	18.8 ± 3.0(15.5–22.0)	23 (21–26)	22.2 (16.8–27.9)	31.7 ± 5.6 (23.4–40.0)	–	41 (36–46)
c'	1.0 ± 0.2(0.9–1.2)	–	–	0.7 ± 0.1 (0.5–1.0)	–	–
V%	90 ± 2(88–92)	91–92	92.2 (91.1–93.7)	93.2 ± 0.6 (92.0–94.1)	90–94	95 (94–95.5)
VL/VB	1.3 ± 0.1(1.2–1.5)	1.1–1.2	1.2 (0.9–1.3)	1.2 ± 0.1 (1.0–1.4)	0.9–1.8	0.8–1.0
R	124 ± 3(120–128)	122 (119–125)	119 (111–127)	111 ± 2.9 (104–115)	100–133	150 (143–157)
Rex	32 ± 1(31–33)	31 (29–34)	31 (30–34)	30 ± 1.2 (27–32)	28–39	(48–50)
RV	12 ± 1(11–12)	12.8 (11–14)	11 (9–12)	9 ± 2.6 (8–10)	7–13	(9–10)
RVan	5 ± 1(4–5)	5 (4–7)	2 (1–3)	4.6 ± 0.5 (4–5)	1–7	3
Ran	7 ± 1(6–8)	6.8 (5–8)	7 (6–9)	4.4 ± 0.5 (4–5)	5–8	(5–7)
Stylet	75 ± 2(72–77)	68 (66–72)	64 (62–71)	65 ± 2.9 (59–69)	74–91	95 (90–97)
Stylet%L	17.5 ± 0.9(16.5–18.3)	–	11.8 (10.6–14.5)	12.3 ± 0.7 (10.9–13.9)	–	–
Stylet%Oes	72 ± 3(68–75)	–			–	–
Pharynx length	104 ± 7(99–113)	–	125 (112–137)	114 ± 4.6 (107–121)	–	–
Max. body diam. (MBD)	38 ± 3(35–41)	–	50 (41–59)	45 ± 5.3 (35–51)	–	–
Vulval body diam. (VBD)	28 ± 3(26–32)	–	–	30 ± 2.0 (26–33)	–	–
Vulva to tail tip (VL)	38 ± 5(31–42)	–	–	36 ± 3.6 (29–41)	–	–
Anal body diam. (ABD)	23 ± 2(20–25)	–	–	23.3 ± 2.0 (20.0–27.0)	–	–
Tail length	24 ± 6(19–30)	–	25 (20–31)	17.2 ± 3.6 (12.0–23.0)	–	–
Ovary length	185 ± 21(155–206)	–	–	–	–	–
Anterior end to nerve ring	83 ± 6(79–91)	–	–	–	–	–

Measurements are in μm and in the form: mean ± s.d. (range).

#### Morphological characterisation

**Figure 1:**
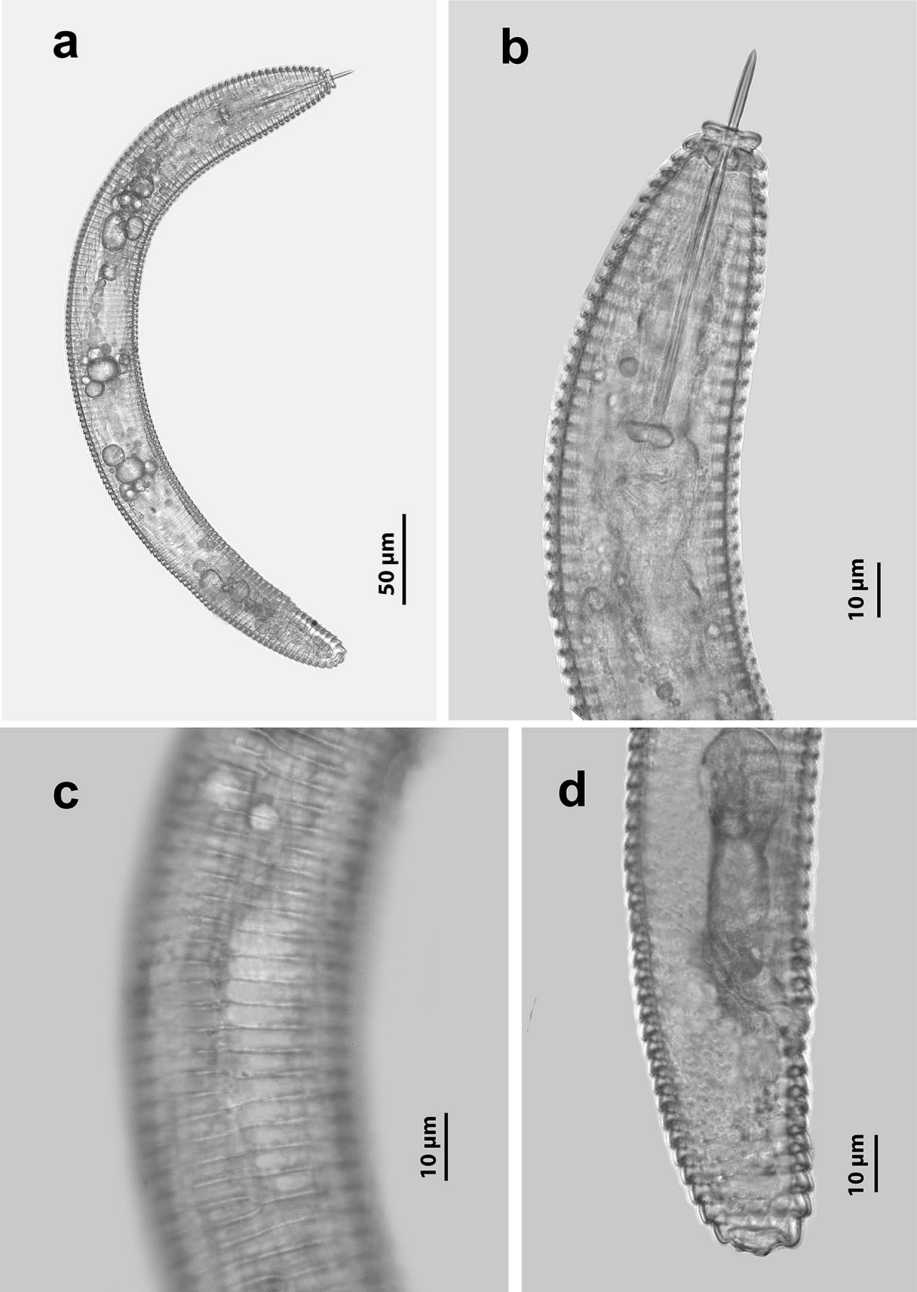
Female of *Criconemoides myungsugae* from Vietnam. (**a)** Entire body. (**b)** Anterior end region. (**c**) Lateral field region. (**d**) Posterior end region.

Females of *Criconemoides myunsugae* recovered from Vietnam are characterised by having a ventrally curved body (Fig. 1a); retrorse annuli with lateral field marked by discontinuous breaks and anastomoses of transverse striae; first lip annulus forming an uninterrupted disc (Fig. 1b); robust and straight stylet with anchor-shaped knobs; typical pharynx of the genus, with fused procorpus and metacorpus, a large valve, a swollen and offset basal bulb from narrowed isthmus (Fig. 1b); small and oval-shaped spermatheca without sperm; monodelphic-prodelphic ovary; closed vulva, flat or slightly protruding above body contour (Fig. 1c); and broadly rounded or slightly tapering tail (Fig. 1d).

#### Morphological relationship

According to the identification key provided by Geraert^1^, the Vietnamese *Criconemoides* population is most similar to *Criconemoides morgensis* and *Criconemoides myungsugae*. However, our nematode population can be clearly distinguished from *Criconemoides morgensis* by first annulus (disc-like *vs* not disc-like), spermatheca (empty *vs* filled with sperm), smaller body length (395–468 *vs* 510–700 µm), smaller stylet length (72–77 *vs* 74–91 µm), and other measurements (see Table 1). Besides, the morphology of our nematode population is in agreement with the original and redescription of the type population of *Criconemoides myungsugae*, except for the slightly longer stylet (72–77 *vs* 66–72 µm) and smaller c value (15.5–22 *vs* 21–26)^28,30^. Furthermore, our population of *Criconemoides* species also showed very high similarity compared to other populations of *C. myungsugae* from Iran, China, Switzerland and the US (Table 1). Therefore, our nematode population is identified morphologically as *C. myungsugae*.

#### Molecular characterisation and relationship

##### Characterisation of 18S rRNA region

**Figure 2:**
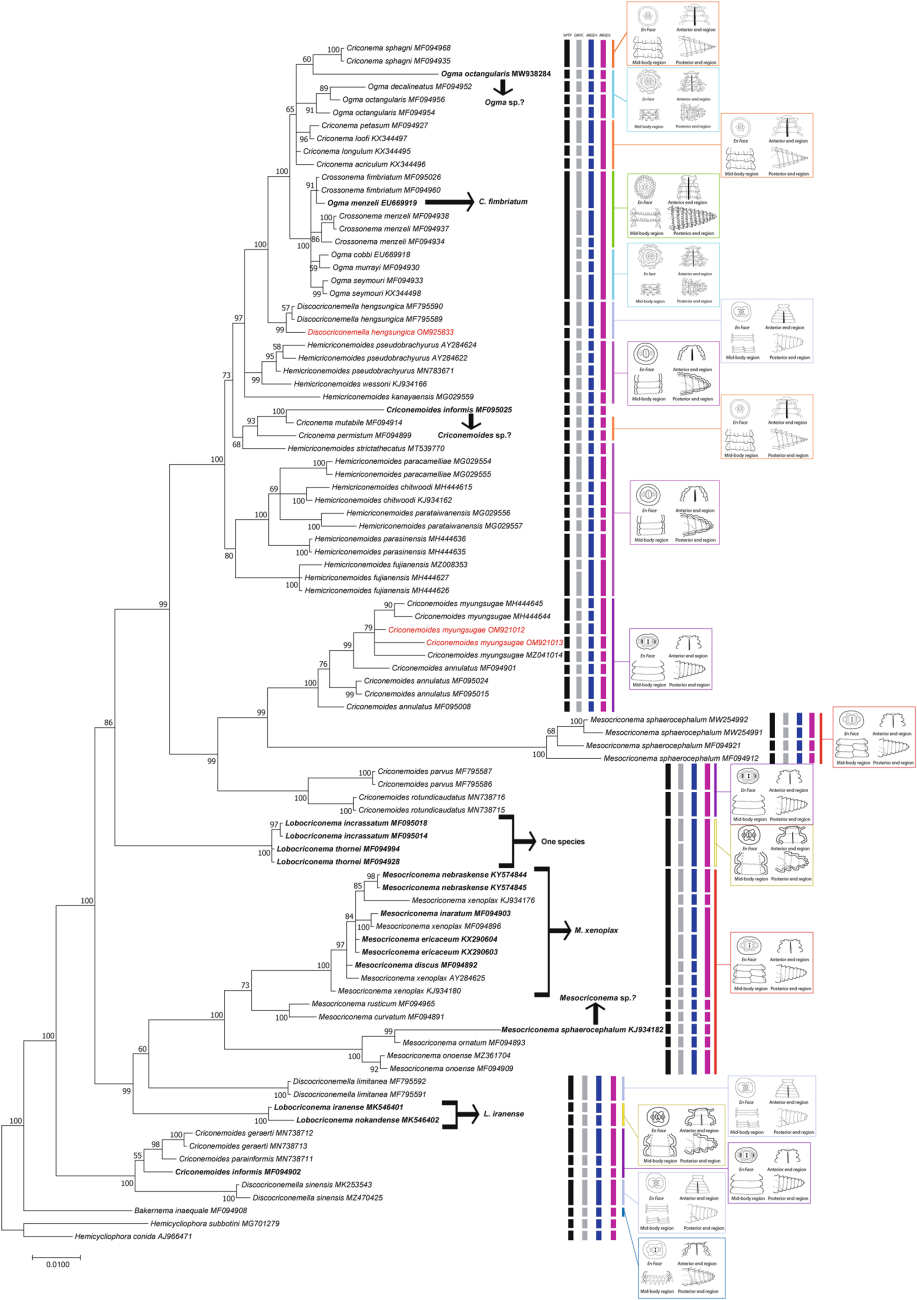
Phylogenetic tree generated from 18S rRNA sequences under GTR + G + I model. Posterior probability (in percentage) is given alongside each node. Sequences of nematode populations from Vietnam are marked in red color. Typical cuticular markings have been assigned to studied genera.

Two 18S rRNA sequences of 837 bp length were obtained for *C. myungsugae* from Vietnam. The intraspecific variation between our sequences was 1% (6 bp difference). Our 18S rRNA sequences were 98.6–99.6% similar (5–14 bp difference) to the sequences of *C. myungsugae* from GenBank (accession number: MZ041014, MH444645, MH444644). Strikingly, they were also 98.8–99.3% similar (only 6–10 bp difference) to the 18S rRNA sequences of *C. annulatus* from GenBank (MF095008, MF095024, MF095015, MF094901). The phylogenetic tree based on 18S rRNA sequences showed that the sequence of *C. myungsugae* from Vietnam was embedded in a poorly-supported clade of *C. myungsugae* (PP 0.79), and in a maximally-supported (PP 1), clade of both *C. myungsugae* and *C. annulatus* (Fig. 2).

However, only one species-delimitation result (ABGD2) recognised one of our 18S rRNA sequences as being conspecific with *C. myungsugae* (MH444645, MH444644). Interestingly, despite the fact that our two 18S sequences were from the same population, two species-delimitation results (GMYC and ABGD1) indicated all five 18S rRNA sequences of *C. myungsugae* (including our sequences) to be five separated species (Fig. 2).

##### Characterisation of D2-D3 of 28S rRNA region

**Figure 3:**
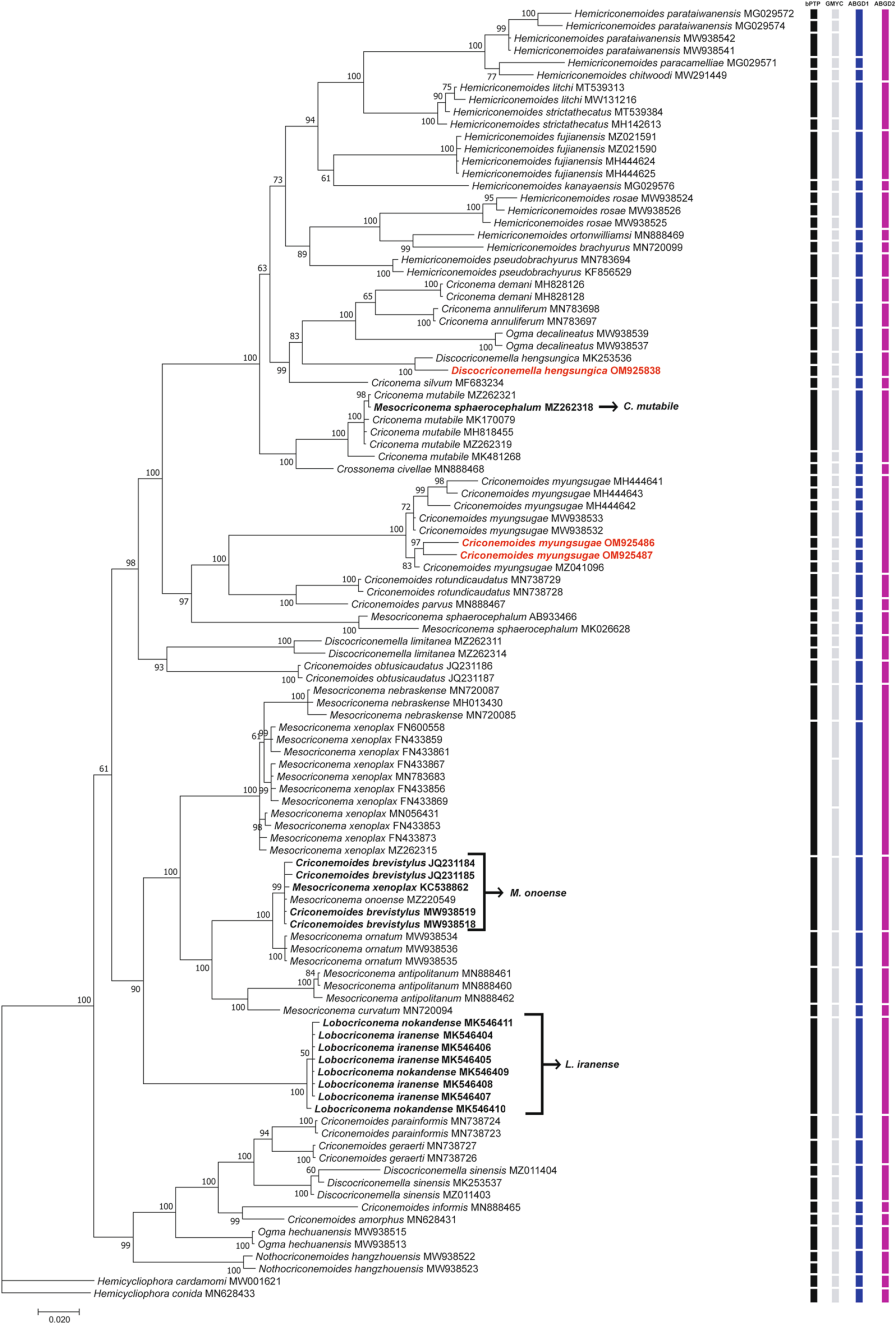
Phylogenetic tree generated from D2-D3 of 28S rRNA sequences under GTR + G + I model. Posterior probability (in percentage) was given next to each node. Sequences of nematode populations from Vietnam were marked by red color.

Two D2-D3 sequences of *C. myungsugae* from Vietnam were obtained with 3% intraspecific variation (721-742 bp long). These sequences were found to be most similar to the sequences of *C. myungsugae* (MZ041096, MW938533, MW938532, MH444643, MH444641, and MH444642) with only 94.9–97.7% similarity (17–34 bp difference). Despite these relatively large differences, the phylogenetic tree based on D2-D3 sequences showed that the sequences of *C. myungsugae* from Vietnam are imbedded in a maximally supported *C. myungsugae* clade, which has a sister relationship to the sequences of *Criconemoides rotundicaudatus* (MN738729, MN738728) and *Criconemoides parvus* (MN888467) (Fig. 3). Conspecificity of all *C. myungsugae* sequences was supported by only one ABGD species-delimitation result (ABGD2), while bPTP, GMYC, and ABGD1 surprisingly recognised the eight *C. myungsugae* sequences as seven different species (Fig. 3).

##### Characterisation of *COI* mtDNA region

**Figure 4:**
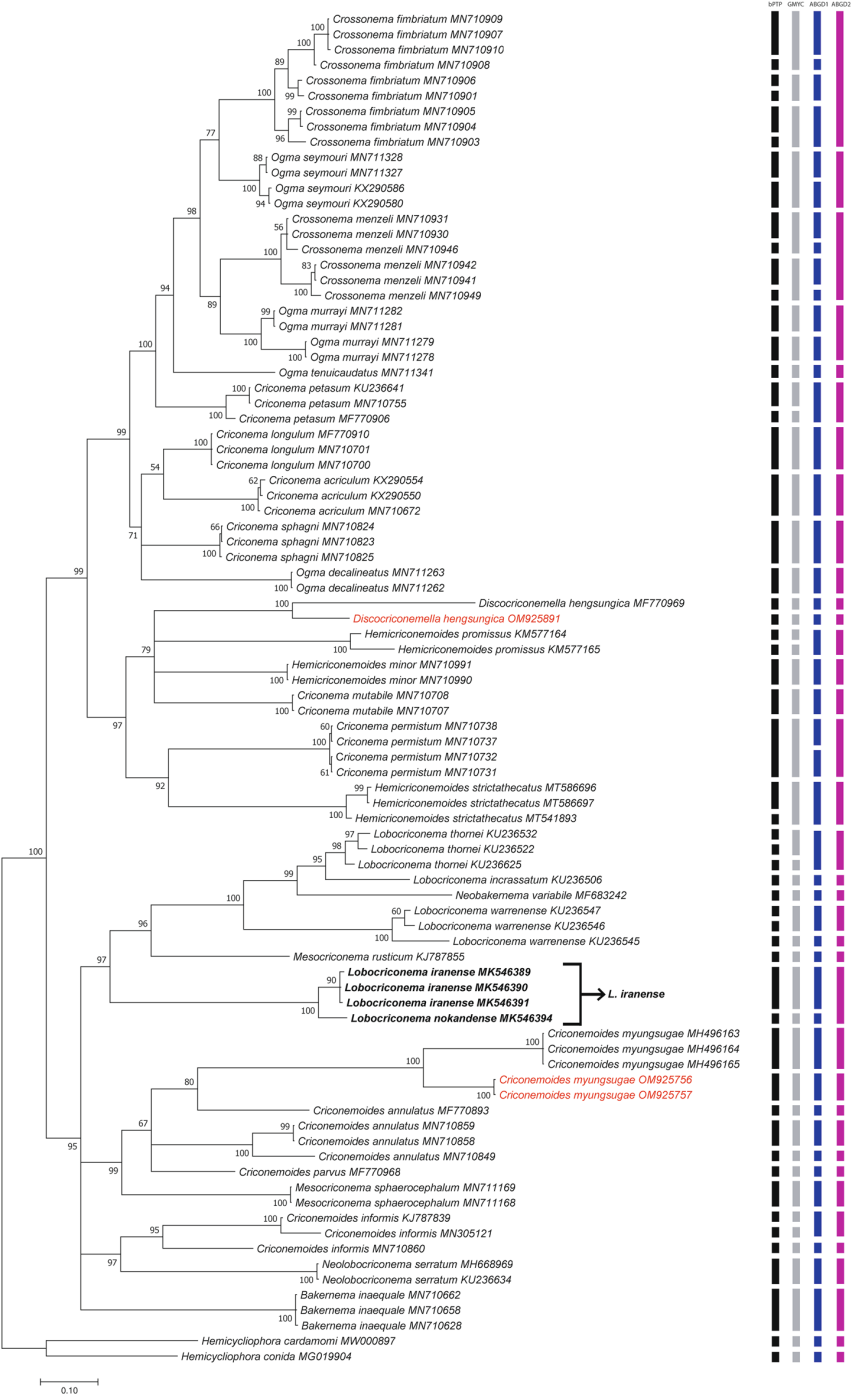
Phylogenetic tree generated from *COI* mtDNA sequences under GTR + G + I model. Posterior probability (in percentage) was given next to each node. Sequences of nematode populations from Vietnam were marked by red color.

Two obtained *COI* sequences of *C. myungsugae* from this study were 439–440 bp long without intraspecific sequence variation. They were only 89% similar (46 bp difference) to the closest sequences of *C. myungsugae* (MH496163, MH496164, MH496165). The resulting *COI* phylogenetic tree showed that the sequences of *C. myungsugae* from Vietnam have a maximal supported sister relationship to the sequence of *C. myungsugae* from GenBank and the *C. myungsugae* clade has a not well-supported sister relationship to one sequence of *C. annulatus* (MF770893), making sequences of *C. annulatus* paraphyletic group (Fig. 4). Remarkably, all four species-delimitation results indicated the two *COI* sequences of *C. myungsugae* of Vietnam as a different species as *C. myungsugae* from GenBank (Fig. 4).

### Characterisations of Discocriconemella hengsungica Choi & Geraert, 1975 from Vietnam

#### Measurements

All measurements of *Discocriconemella hengsungica* from Vietnam are provided in Table 2.

**Table 2:** Morphometrics of *Discocriconemella hengsungica* from Vietnam and the world.

Character	*Discocriconemella hengsungica*		
Source and locality	This study Vietnam	Choi & Geraert^27^ Korea	Maria et al., 2018 China
n	10	5	15
L	369 ± 13 (356–381)	285–315	333 ± 19 (308–383)
a	9.2 ± 0.4 (8.8–9.8)	8.2–9.8	9.4 ± 1.0 (8.2–11.5)
b	2.7 ± 0.03 (2.6–2.7)	2.3–2.6	2.5 ± 0.1 (2.3–2.8)
c	12.0 ± 0.8 (11.2–13.1)	–	19.6 ± 3.0 (15.2–25.7)
c'	1.1 ± 0.06 (1–1.1)	–	0.7 ± 0.1 (0.5–0.8)
V%	89 ± 1 (88–90)	87–90	89 ± 0.8 (88–90)
VL/VB	1.2 ± 0.08 (1.1–1.3)	–	1.1 ± 0.1 (1.0–1.3)
R	92 ± 2.4 (89–94)	82–92	91 ± 2.2 (88–94)
Rex	27 ± 1 (26–28)	–	34 ± 1.6 (30–36)
RV	11 ± 1(10–12)	13	9.8 ± 0.4 (9.0–10.0)
RVan	2 ± 1(2–3)	–	5.2 ± 0.8 (4.0–6.0)
Ran	9 ± 1(8–10)	–	4.7 ± 0.7 (4.0–6.0)
Stylet	107 ± 2 (105–110)	104–108	107 ± 3.4 (100–114)
Stylet%L	29 ± 0.6 (28–29)	–	32 ± 1.7 (29–35)
Stylet%Oes	77 ± 1.7 (75–79)	–	–
Pharynx length	139 ± 5 (133–143)	–	132 ± 3.3 (126–138)
Max. body diam. (MBD)	40 ± 1.9 (39–43)	–	36 ± 2.7 (30–39)
Vulval body diam. (VBD)	32 ± 1.5 (31–34)	–	32 ± 1.9 (28–34)
Vulva to tail tip (VL)	39 ± 2.1(36–41)	–	36 ± 3.2 (31–43)
Anal body diam. (ABD)	29 ± 1.3(28–31)	–	26.5 ± 2.1 (22.5–29.4)
Tail length	31 ± 1.5(29–32)	–	17.4 ± 2.8 (12.0–21.8)
Anterior end to nerve ring	121 ± 3 (117–125)	–	–
Anterior end to Secretory-excretory pore	110 ± 1.8 (108–112)	–	–

Measurements are in μm and in the form: mean ± s.d. (range).

#### Morphological characterisation

**Figure 5:**
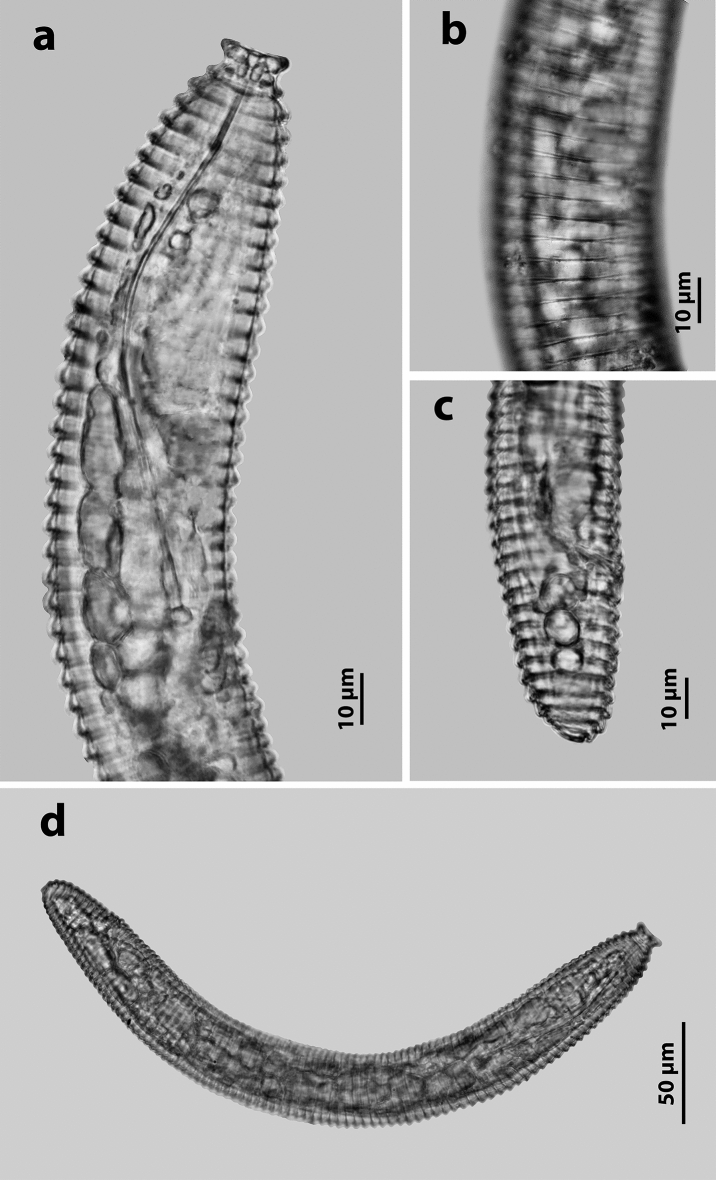
Female of *Discocriconemella hengsungica* from Vietnam. (**a**) Anterior end region. (**b**) Lateral field region. (**c**) Posterior end region. (**d**) Entire body.

Females of *Discocriconemella hengsungica* from Vietnam are characterised by having a body curved ventrally body after heat-fixing (Fig. 5d); lateral field without anastomoses (Fig. 5b); first lip annulus forming a disc-like followed by a long cylindrical neck (Fig. 5a); long, slender, and curved stylet with anchor-shaped knobs (Fig. 5a); oval-shaped spermatheca with or without sperm; outstretched and monodelphic-prodelphic ovary; closed vulval (Fig. 5c); broadly rounded tail with indistinct anus (Fig. 5c).

#### Morphological relationship

The morphology of *Discocriconemella* species in this study was most similar to *D. hengsungica*, *D. retroversa*, and *D. spermata* according to the identification key of Geraert^1^. Our nematode population can be differentiated from *D. retroversa* by longer body length (356–381 vs 250–310 µm), longer stylet length (105–110 vs 88–97 µm), smaller R value (89–94 vs 94–105), smaller Rex value (26–28 vs 30–32), anastomoses at lateral field (absent vs present), and male (absent vs present). The population of *D. hengsungica* from Vietnam differs from *D. spermata* by longer body length (356–381 vs 270–360 µm), smaller RVan value (2–3 vs 4–7), larger Ran value (8–10 vs 5–9), anastomoses at lateral field (absent vs present), and male (absent vs present). The morphology of *D. hengsungica* from Vietnam is only different from the original description of Choi et al.^28^ by slightly larger body length (356–381 vs 285–315 µm) (Table 2).

#### Molecular characterisation and relationship

##### Characterisation of 18S rRNA region

The 18S rRNA sequence of *D. hengsungica* from Vietnam was 887 bp long. Our sequence was 99.7% similar (3 bp difference) to the other sequences of *D. hengsungica* (MF795590, MF795589). The phylogenetic tree based on 18S rRNA sequences showed that all sequences of *D. hengsungica* (including our sequence) formed a separated clade with 0.99 PP support (Fig. 2). Although ABDG2 recognised our 18S rRNA sequence to be conspecific with *D. hengsungica*, the first three species-delimitation results (bPTP, GMYC, ABDG1) separated our 18S rRNA sequence as a different species (Fig. 2).

##### Characterization of D2-D3 of 28S rRNA region

The D2-D3 sequence of *D. hengsungica* from Vietnam was 749 bp long and most similar to of *D. hengsungica* (MK253536) with 96% similarity (21 bp difference). The resulting D2-D3 tree topology indicated a maximally supported relationship of all *D. hengsungica* sequences (Fig. 3). Despite the remarkably dissimilarity between the two *D. hengsungica* sequences, two ABGD species-delimitation results confirmed their conspecificity. However, bPTP and GMYC species-delimitation methods recognised these sequences as two different species (Fig. 3).

##### Characterisation of *COI* mtDNA region

The *COI* sequences of *D. hengsungica* obtained from this study was 359 bp long and only 88% similar (44 bp difference) to the sequence of *D. hengsungica* from GenBank (MF770969). Nevertheless, the aforementioned sequences were placed in a maximally supported sister relationship (Fig. 4). Similar to the case of *C. myungsugae*, all four species-delimitation results recognised these sequences as two separated species (Fig. 4). However, several studies indicated that the molecular variation of *COI* can be relatively large between different populations of a single species^27,31^. Consequently, all the species-delimitation methods used in this study recognised much higher number of molecular species based on *COI* than the number of corresponding morpho-species (Table 3).

**Table 3:** Comparing the number of established species and molecular species-delimitation results according to different species-delimitation methods of Criconematidae with sequence representatives in GenBank.

Gene region	Number of species					
	Originally considered	After our revision	bPTP	GMYC	ABGD1	ABGD2
18S rRNA	51	47	55	71	69	47
D2-D3 of 28S rRNA	42	40	56	56	47	35
*COI* mtDNA	31	31	55	46	47	37

#### Remarks on molecular variation and phylogenetic relationship of studied species

**Figure 6:**
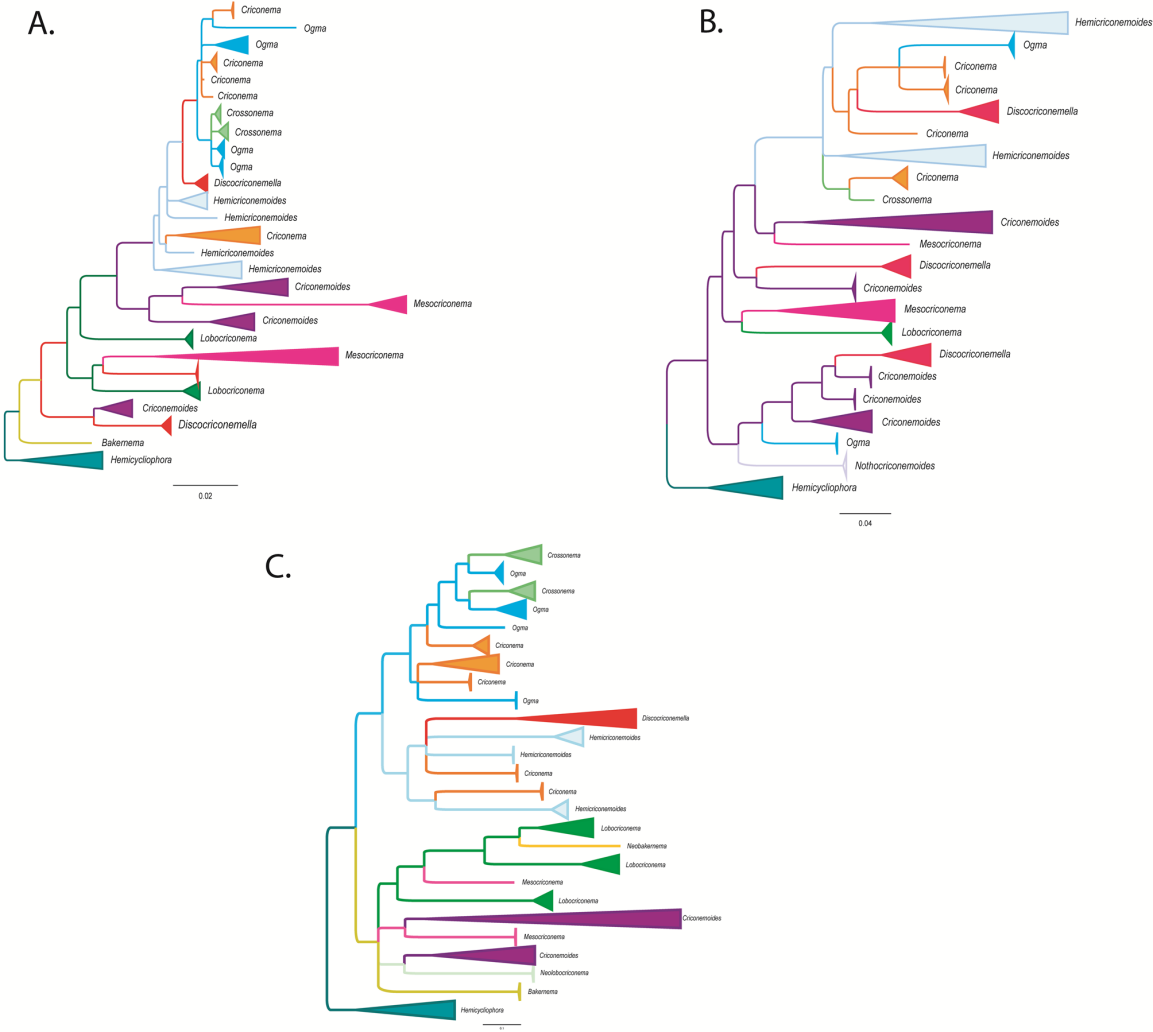
Simplified phylogenetic trees showing relationships between genera in Criconematidae in this study. (**A**) 18S tree; (**B**) D2-D3 tree; (**C**) *COI* tree.

It is clear that phylogenetic positions of certain genera in the family Criconematidae are not well-resolved, and that almost none of the studied genera form monophyletic groups (Fig. 6). On the tree constructed from 18S rRNA sequences, virtually all genera, including *Criconema*, *Ogma*, *Crossonema*, *Discocriconemella*, *Hemicriconemoides*, *Criconemoides*, *Mesocriconema*, and *Lobocriconema*, appeared in at least two different clades (Fig. 6). No conclusions can be drawn regarding *Bakernema* since only the sequences of one species, *Bakernema inaequale*, are available (Fig. 2). Similarly, the phylogenetic trees created from D2-D3 and *COI* sequences also show the non-monophyly of the above genera, albeit relatively weakly-supported, since the posterior probabilities at crucial nodes are relatively low (Figs. 2, 3, 4, 6). Some genera, including *Nothocriconemoides* and *Neolobocriconema*, appear to be monophyletic; most likely because of a more limited representation of sequences compared to the 18S analyses (Figs. 3, 4).

To confirm the monophyly hypothesis of different genera in our study, SH and AU tests were employed. For the 18S dataset, SH and AU tests rejected the monophyly hypothesis of seven genera, including *Criconema*, *Ogma*, *Crossonema*, *Discocriconemella*, *Hemicriconemoides*, *Criconemoides*, and *Lobocriconema* (p < 0.001). Although the Log-likelihood of unconstrained tree was much higher than the constrained tree of *Mesocriconema* as monophyletic (-9001.42 vs -15074.48), this difference is not statistically significant as indicated by the SH and AU tests (p > 0.05). For the D2-D3 dataset, SH and AU tests also rejected the monophyly hypothesis of six genera, including *Criconema*, *Ogma*, *Discocriconemella*, *Criconemoides*, and *Lobocriconema* (p < 0.001), and *Hemicriconemoides* (p < 0.005). Similarly, SH and AU tests did not reject the monophyly hypothesis of *Mesocriconema* in 28S dataset. For the *COI* dataset, SH and AU tests rejected the monophyly hypothesis of four genera, including *Criconemoides* (p < 0.01), *Hemicriconemoides* (p < 0.01), *Mesocriconema* (p < 0.05), and *Ogma* (p < 0.05).

Interestingly, intraspecific variations of many species in Criconematidae are relatively high (up to 2.6%, 6.5%, and 12% for 18S, D2-D3, and *COI* sequences, respectively), e.g. variations in sequences of *Discocriconemella hengsungica*, *Hemicriconemoides pseudobrachyurus*, *Hemicriconemoides chitwoodi*, *Hemicriconemoides parataiwanensis*, *Criconemoides myungsugae*, *Criconemoides annulatus*, *Mesocriconema sphaerocephalum*, *Discocriconemella limitanea*, and *Discocriconemella sinensis*. Therefore, the four molecular species-delimitation results that were used did often not correspond to the corresponding species demarcation based on morphology (Figs. 2, 3, 4).

The high molecular variations in the family Criconematidae alongside our tree- and distance-based molecular species-delimitation results and other available data suggest that 40 sequences, of 14 different species, on GenBank are required to be either corrected or re-considered (Table 4). All sequences that were used in our study with the indication of accession numbers and comments on sequence status are also provided (Table S1).

**Table 4:** Sequences in this study that were mislabeled, misidentified, or need revision.

No	Species name	Accession number			Remarks	Decision
		18S	28S	COI		
1.	*Lobocriconema iranense*	MK546401	MK546404 MK546406 MK546405 MK546408 MK546407	MK546389 MK546390 MK546391	- These two species are morphologically and molecularly very similar - 28S phylogenetic tree reveals that these sequences indeed belong two one species - Species delimitation results prove that the hypothesis these two species are conspecific cannot be rejected.	These two species should be attributed to a single species
2.	*Lobocriconema nokandense*	MK546402	MK546410 MK546411 MK546409	MK546394		
3.	*Mesocriconema sphaerocephalum*	KJ934182			- This sequence was provided by Zeng, et al.^32^ without morphological data - This sequence is clearly separated from other 18S sequences of *Mesocriconema sphaerocephalum*(MW254991, MW254992, MF094921, MF094912; supported by morphological and molecular data^33^)	This sequence was misidentified
4.	*Mesocriconema sphaerocephalum*		MZ262318		- Phylogenetic analyses and all species delimitation methods indicate that this sequence is conspecific to *Criconema mutabile*(MZ262321, MK170079, MH818455, MZ262319, MK481268; supported by several independent studies including supporting morphological data^34^)	This sequence represents *Criconema mutabile*
5.	*Criconemoides brevistylus *		JQ231184 JQ231185 MW938519 MW938518		- The variations between these sequences and *Mesocriconema onoense* (MZ220549) are only 0.1-1.3% (1-9 bp difference) and the bPTP species-delimitation method indicates that these sequences belong to the same species	These sequences represent *Mesocriconema onoense*
6	*Mesocriconema xenoplax *		KC538862			
7.	*Mesocriconema discus *	MF094892			- This sequence is not linked with morphological data and is 98.8-99.6% similar (9-12 bp difference) and clusters with the sequences of *M. xenoplax* (KJ934176, KJ934180, MF094896, AY284625) on 18S tree - bPTP and ABGD species delimitation methods indicate this sequence as conspecific with sequences of* Mesocriconema xenoplax*	This sequence represents *Mesocriconema xenoplax*
8.	*Mesocriconema ericaceum *	KX290604 KX290603			- Powers, et al.^35^ stated that this species superficially morphologically resembles *M*. *xenoplax * - These sequences are 98.8-99.8% similar (only 2-11 bp difference) to sequences of *M. xenoplax *(KJ934176, KJ934180, MF094896, AY284625) - bPTP and ABGD species delimitation methods recognised these sequences as conspecific with *Mesocriconema xenoplax*	These sequences represent *Mesocriconema xenoplax*
9.	*Mesocriconema nebraskense*	KY574844 KY574845	MN720085 MH013430 MN720087		- Although authors stated that* M. nebraskense *is morphologically very similar to *M. curvatum*, body length of *M. nebraskense *is more similar to *M. xenoplax *(393-606 *vs *404-620 µm) than to *M. curvatum *(393-606 *vs *303-452 µm)^36,37^ - The two identical 18S sequences of *M. nebraskense *are 99.1-99.9% similar (only 1-16 bp difference) to sequences of *M. xenoplax*, *M. inaratum*, *M. ericaceum*, and *M. discus* - bPTP species delimitation method recognises 18S sequences of *M. nebraskense* as conspecific with sequences of* Mesocriconema xenoplax*	These sequences represent *Mesocriconema xenoplax*
10.	*Mesocriconema inaratum *	MF094903			- The 18S sequence of *M. inarata *(MF094903) is identical to the sequences of *Mesocriconema xenoplax* (AY919192 and MF094896) from the type location provided by the same authors^38^ - bPTP and ABGD species delimitation methods indicate this sequence and those of* Mesocriconema xenoplax* as conspecific	This sequence represents *Mesocriconema xenoplax*
11.	*Lobocriconema thornei *	MF094994 MF094928			- These sequences were provided by Powers, et al.^11^ without morphological data - The 18S sequences of *Lobocriconema incrassatum *and * Lobocriconema thornei *are 99.9% similar (only 2 bp difference) - Three out of four species-delimitation results indicate these sequences as conspecific (Fig. 2)	These sequences belong to a single species and morphological data of these nematode populations need to be reviewed
12.	*Lobocriconema incrassatum *	MF095018 MF095014				
13.	*Criconemoides informis *	MF095025 MF094902			- Powers, et al.^11^ provided these two 18S sequences of *Criconemoides informis* without morphological data - These sequences are distantly separated from each other and are 61 bp different	At least one of these sequences must be mislabelled
14.	*Ogma octangularis *	MW938284 MF094956 MF094954			- These sequences are available on GenBank without morphological data - The two 18S sequences of *Ogma octangularis *(MF094956, MF094954) were published in studies of Powers, et al.^11^, while the other (MW938284) is available on GenBank via a direct submission - Four species-delimitation results indicate that these sequences represent at least two different species	Other taxonomical data are needed to confirm the status of these sequences
15.	*Ogma menzeli *	EU669919			- This sequence is available on GenBank without morphological data - This sequence is placed together with sequences of *Crossonema fimbriatum *(accession number: MF095026, MF094960; provided by Powers, et al.^11^) in a single clade (99.8-99.9% similarity and only 2-3 bp difference), separated (differed by 8-10 bp) from three other sequences of *Crossonema menzeli* (accession number: MF094934, MF094937, MF094938; Powers, et al.^11^) - Two species-delimitation results (GMYC and ABGD1) indicate that the sequence of *Ogma menzeli* (EU669919) and the sequences of *Crossonema fimbriatum *(MF095026, MF094960 are conspecific	This sequence represents* Crossonema fimbriatum*

## Discussion

The classification of criconematid species has until now been based on morphology alone (especially cuticular markings) and is widely considered to be volatile^1,9^. Although Subbotin et al.^10^ observed the monophyly of the genera *Mesocriconema*, *Hemicriconemoides*, and *Criconema* in the suborder Criconematina based on D2-D3 data, our analysis based on more recent 18S, D2-D3, and *COI* sequences indicated none of these genera as being monophyletic. Likewise, the phylogenetic analysis of Powers et al.^11^, based on 18S sequences, showed that *Lobocriconema* is a monophyletic group and *Criconemoides* is paraphyletic. However, our 18S, D2-D3, and *COI* tree topologies indicated that *Lobocriconema* and *Criconemoides* are also both polyphyletic groups (Figs. 1, 2, 3). Furthermore, the SH and AU tests based on 18S, D2-D3, and/or *COI* datasets rejected the monophyly hypothesis of eight genera in Criconematidae, i.e. *Criconema*, *Ogma*, *Crossonema*, *Discocriconemella*, *Hemicriconemoides*, *Criconemoides*, *Mesocriconema*, and *Lobocriconema*. Thus, based on current and former molecular analyses, none of the generic groupings within the family Criconematidae can be supported, except for those genera with limited available sequences on GenBank (*Bakernema*, *Nothocriconemoides* and *Neolobocriconema*). Our updated molecular analyses suggest that key morphological characters used in the classification of Criconematidae have resulted from convergent evolution. For example, the species of genus *Hemicriconemoides* with double cuticle did not form a monophyletic group; the large labial disc in *Discocriconemella* appears to have evolved at least three times independently, in agreement with previous studies^11,39^; the very coarse annuli typical for *Lobocriconema* appeared in two distant clades; species belonging to *Criconemoides* (without cuticular outgrowths) appear in three different clades; the genera *Criconema* (cuticle with continuous fringe or longitudinal rows) and *Ogma* (cuticle with numerous appendages, arranged in 6–26 longitudinal rows) appear in at least two different phylogenetic conditions (Figs. 2, 3, 4, 6).

In this study, we also revealed the remarkable molecular variations in several criconematid species, a case in point being our population of *C. myungsugae*. This nematode population agrees morphologically with the type population and other descriptions. Although 18S, D2-D3, and *COI* analyses indicated its close relation with other populations of *C. myungsugae*, remarkable molecular variations between sequences of *C. myungsugae* can be observed. Intrapopulation variations of *C. myungsugae* from Vietnam for 18S, D2-D3, and *COI* sequences were respectively 0.9%, 3.7%, and 0%, and they differed by 0.5–1.1%, 2.3–5.1%, and 10.9–11% respectively to the sequences of *C. myungsugae* from GenBank. *Criconemoides myungsugae* from Vietnam was, therefore, also appointed as a distinct species (or may even be appointed as several species) by several of the molecular species-delimitation approaches that were employed. Similarly, high molecular variations were also recorded among 18S, D2-D3, and *COI* sequences of *D. hengsungica* (0.3%, 4.3%, and 12.3%, respectively) that also resulted in putative species splitting, according to several of the species-delimitations methods used. Other authors^35,36,40^ considered such molecular differences sufficient to establish the description of new species. However, given the high intraspecific/intrapopulation variations observed in this study, the distinction between intra- and interspecific variations (the essence of molecular species-delimitations) is not unequivocal. This is also reflected in the contradictions between the used species-delimitation methods, with only ABGD results (ABGD1 and ABGD2) appointing the respective populations of *C. myungsugae* and *D. hengsungica* as conspecific according to 18S and D2-D3 analyses (Figs. 2, 3).

It is notable that virtually all the molecular species-delimitation approaches used in this study resulted in a considerably larger number of putative species compared to the number of established species (Tables 3, 4). These results agree with other studies indicating that tree-based and distance-based species-delimitation methods tend to recover a higher number of phylogenetic lineages than the number of species originally considered^29,41,42^. Conversely, the ABGD2 and bPTP output appears to be over-conservative when delimitating several *Crossonema*, *Criconema*, and *Ogma* species. For example, the 18S sequences of five species including *Crossonema fimbriatum*, *C. menzeli*, *Ogma cobbi*, *Ogma murrayi*, and *Ogma seymouri* were delineated as a single species. However, the number of putative species detected according to the ABGD2 output remarkably well agreed with the number of established species (after taking the presented suggestions into account), i.e. an identical, 5 less, and 6 additional species in comparison, respectively for the 18S, D2-D3, and *COI* dataset (Table 3, Figs. 2, 3, 4). Nonetheless, despite this attractively convenient-looking match, we cannot simply champion ABGD as the preferred method for molecular species delineation, given the highly inconsistent ABGD outputs (i.e. ABGD1 *vs* ABGD2). We also concur with Hofmann et al.^29^ and Prevot et al.^43^ that to avoid over- or underestimation of molecular species delimitation, a combination of tree-based and distance-based methods should be employed to ensure a more stable taxonomic interpretation. Taken all together, the selection of a single species-delimitation method is not straightforward, and thus, molecular species-delimitation methods should be considered as one of the techniques to be used in comprehensive polyphasic taxonomy. Furthermore, this study also clearly revealed that, for criconematid species, 40 sequences belonging to 14 different species on GenBank have been mislabeled, unlabeled, associated with misidentified sequences, or need to be reviewed, a finding that agrees with previous studies^31,44^. Therefore, both the new species descriptions as well as records of known species must be validated by polyphasic studies which combine analyses of both morphological and molecular approaches. This represents the clearest way forward in order to avoid confusion and to advance nematode taxonomy in general^31,34,41,45–48^.

## Conclusions

Phylogenetic analyses, supported by tree topology tests, based on different gene regions confirmed that virtually all studied criconematid genera (except for those with limited available sequences in GenBank) are not monophyletic groups. Key morphological characters used in the classification of Criconematidae are likely to be the consequence of convergent evolution. This study provides the first report of *Criconemoides myungsugae* and *Discocriconemella hensungica* associated with Vietnamese ginseng, one of the most precious and rare ginseng varieties in the world. Besides, our molecular analyses also revealed the high molecular intraspecific variations, with our nematode populations as cases in point, in many criconematid species that were considered as enough to establish new cryptic species by some nematologists. The combination of different tree- and distance-based molecular species delimitation methods used in our study have helped in the declamation of molecular species boundary more stably. Our polyphasic study also indicated a number of sequences from GenBank must be either revised or reconsidered.

## Supplementary Information

Supplementary Information.

## Data Availability

All data generated or analysed during this study are included in this published article.
