# Supplementary material for: Remarks on phylogeny and molecular variations of criconematid species (Nematoda: Criconematidae) with case studies from Vietnam
Source: Sci Rep. 2022 Sep 1;12:14832. doi: 10.1038/s41598-022-18004-2 (PMC9437051; doi:10.1038/s41598-022-18004-2)
Supplement: Supplementary file 1 — Supplementary Information. [file 41598_2022_18004_MOESM1_ESM.docx]

Table S1. List of all criconematid sequences that were used in this study

| No | Species name | Accession number | | | Comments |
| --- | --- | --- | --- | --- | --- |
|  |  | 18S | 28S | COI |  |
|  | *Bakernema inaequale* | MF094908 |  |  |  |
|  |  |  |  | MN710662 |  |
|  |  |  |  | MN710658 |  |
|  |  |  |  | MN710628 |  |
|  | *Criconema acriculum* | KX344496 |  |  |  |
|  |  |  |  | MN710672 |  |
|  |  |  |  | KX290554 |  |
|  |  |  |  | KX290550 |  |
|  | *Criconema annuliferum* |  | MN783698 |  |  |
|  |  |  | MN783697 |  |  |
|  | *Criconema demani* |  | MH828126 |  |  |
|  |  |  | MH828128 |  |  |
|  | *Criconema longulum* | KX344495 |  |  |  |
|  |  |  |  | MF770910 |  |
|  |  |  |  | MN710701 |  |
|  |  |  |  | MN710700 |  |
|  | *Criconema loofi* | KX344497 |  |  |  |
|  | *Criconema mutabile* | MF094914 |  |  |  |
|  |  |  | MK481268 |  |  |
|  |  |  | MK170079 |  |  |
|  |  |  | MH818455 |  |  |
|  |  |  | MZ262319 |  |  |
|  |  |  | MZ262321 |  |  |
|  |  |  |  | MN710708 |  |
|  |  |  |  | MN710707 |  |
|  | *Criconema permistum* | MF094899 |  |  |  |
|  |  |  |  | MN710738 |  |
|  |  |  |  | MN710737 |  |
|  |  |  |  | MN710732 |  |
|  |  |  |  | MN710731 |  |
|  | *Criconema petasum* | MF094927 |  |  |  |
|  |  |  |  | MF770906 |  |
|  |  |  |  | KU236641 |  |
|  |  |  |  | MN710755 |  |
|  | *Criconema silvum* |  | MF683234 |  |  |
|  | *Criconema sphagni* |  |  | MN710825 |  |
|  |  |  |  | MN710824 |  |
|  |  |  |  | MN710823 |  |
|  |  | MF094968 |  |  |  |
|  |  | MF094935 |  |  |  |
|  | *Criconemoides annulatus* | MF094901 |  |  |  |
|  |  | MF095008 |  |  |  |
|  |  | MF095015 |  |  |  |
|  |  | MF095024 |  |  |  |
|  | *Criconemoides brevistylus* |  | JQ231184 |  | These sequences represent *Mesocriconema onoense* |
|  |  |  | JQ231185 |  |  |
|  |  |  | MW938519 |  |  |
|  |  |  | MW938518 |  |  |
|  | *Criconemoides geraerti* | MN738712 |  |  |  |
|  |  | MN738713 |  |  |  |
|  |  |  | MN738727 |  |  |
|  |  |  | MN738726 |  |  |
|  | *Criconemoides informis* |  |  | MN710860 |  |
|  |  |  |  | KJ787839 |  |
|  |  |  |  | MN305121 |  |
|  |  |  | MN888465 |  |  |
|  |  | MF095025 |  |  | At least one of these sequences must be mislabelled |
|  |  | MF094902 |  |  |  |
|  | *Criconemoides myungsugae* | MZ041014 |  |  |  |
|  |  | MH444645 |  |  |  |
|  |  | MH444644 |  |  |  |
|  |  | OM921012 |  |  |  |
|  |  | OM921013 |  |  |  |
|  |  |  | MZ041096 |  |  |
|  |  |  | MW938533 |  |  |
|  |  |  | MW938532 |  |  |
|  |  |  | MH444642 |  |  |
|  |  |  | MH444641 |  |  |
|  |  |  | MH444643 |  |  |
|  |  |  | OM925486 |  |  |
|  |  |  | OM925487 |  |  |
|  |  |  |  | MH496163 |  |
|  |  |  |  | MH496164 |  |
|  |  |  |  | MH496165 |  |
|  |  |  |  | OM925756 |  |
|  |  |  |  | OM925757 |  |
|  | *Criconemoides obtusicaudatus* |  | JQ231186 |  |  |
|  |  |  | JQ231187 |  |  |
|  | *Criconemoides parainformis* | MN738711 |  |  |  |
|  |  |  | MN738724 |  |  |
|  |  |  | MN738723 |  |  |
|  | *Criconemoides parvus* | MF795587 |  |  |  |
|  |  | MF795586 |  |  |  |
|  |  |  | MN888467 |  |  |
|  |  |  |  | MF770968 |  |
|  | *Criconemoides rotundicaudatus* | MN738716 |  |  |  |
|  |  | MN738715 |  |  |  |
|  |  |  | MN738729 |  |  |
|  |  |  | MN738728 |  |  |
|  | *Crossonema civellae* |  | MN888468 |  |  |
|  | *Crossonema fimbriatum* | MF095026 |  |  |  |
|  |  | MF094960 |  |  |  |
|  |  |  |  | MN710903 |  |
|  |  |  |  | MN710905 |  |
|  |  |  |  | MN710904 |  |
|  |  |  |  | MN710906 |  |
|  |  |  |  | MN710901 |  |
|  |  |  |  | MN710908 |  |
|  |  |  |  | MN710909 |  |
|  |  |  |  | MN710907 |  |
|  |  |  |  | MN710910 |  |
|  | *Crossonema menzeli* | MF094934 |  |  |  |
|  |  | MF094938 |  |  |  |
|  |  | MF094937 |  |  |  |
|  |  |  |  | MN710931 |  |
|  |  |  |  | MN710930 |  |
|  |  |  |  | MN710946 |  |
|  |  |  |  | MN710949 |  |
|  |  |  |  | MN710942 |  |
|  |  |  |  | MN710941 |  |
|  | *Discocriconemella hengsungica* | MF795590 |  |  |  |
|  |  | MF795589 |  |  |  |
|  |  | OM925833 |  |  |  |
|  |  |  | MK253536 |  |  |
|  |  |  | OM925838 |  |  |
|  |  |  |  | MF770969 |  |
|  |  |  |  | OM925891 |  |
|  | *Discocriconemella limitanea* | MF795592 |  |  |  |
|  |  | MF795591 |  |  |  |
|  |  |  | MZ262311 |  |  |
|  |  |  | MZ262314 |  |  |
|  | *Discocriconemella sinensis* | MK253543 |  |  |  |
|  |  |  | MK253537 |  |  |
|  |  | MZ470425 |  |  |  |
|  |  |  | MZ011403 |  |  |
|  |  |  | MZ011404 |  |  |
|  | *Hemicriconemoides brachyurus* |  | MN720099 |  |  |
|  | *Hemicriconemoides chitwoodi* |  | MW291449 |  |  |
|  |  | MH444615 |  |  |  |
|  |  | KJ934162 |  |  |  |
|  | *Hemicriconemoides fujianensis* | MZ008353 |  |  |  |
|  |  | MH444627 |  |  |  |
|  |  | MH444626 |  |  |  |
|  |  |  | MZ021591 |  |  |
|  |  |  | MZ021590 |  |  |
|  |  |  | MH444624 |  |  |
|  |  |  | MH444625 |  |  |
|  | *Hemicriconemoides kanayaensis* | MG029559 |  |  |  |
|  |  |  | MG029576 |  |  |
|  | *Hemicriconemoides litchi* |  | MT539313 |  |  |
|  |  |  | MW131216 |  |  |
|  | *Hemicriconemoides minor* |  |  | MN710991 |  |
|  |  |  |  | MN710990 |  |
|  | *Hemicriconemoides ortonwilliamsi* |  | MN888469 |  |  |
|  | *Hemicriconemoides paracamelliae* | MG029554 |  |  |  |
|  |  | MG029555 |  |  |  |
|  |  |  | MG029571 |  |  |
|  | *Hemicriconemoides parasinensis* | MH444636 |  |  |  |
|  |  | MH444635 |  |  |  |
|  | *Hemicriconemoides parataiwanensis* | MG029556 |  |  |  |
|  |  | MG029557 |  |  |  |
|  |  |  | MG029572 |  |  |
|  |  |  | MG029574 |  |  |
|  |  |  | MW938542 |  |  |
|  |  |  | MW938541 |  |  |
|  | *Hemicriconemoides promissus* |  | KF856529 |  |  |
|  |  |  |  | KM577164 |  |
|  |  |  |  | KM577165 |  |
|  | *Hemicriconemoides pseudobrachyurus* | MN783671 |  |  |  |
|  |  | AY284624 |  |  |  |
|  |  | AY284622 |  |  |  |
|  |  |  | MN783694 |  |  |
|  | *Hemicriconemoides rosae* |  | MW938525 |  |  |
|  |  |  | MW938524 |  |  |
|  |  |  | MW938526 |  |  |
|  | *Hemicriconemoides strictathecatus* | MT539770 |  |  |  |
|  |  |  | MH142613 |  |  |
|  |  |  | MT539384 |  |  |
|  |  |  |  | MT541893 |  |
|  |  |  |  | MT586696 |  |
|  |  |  |  | MT586697 |  |
|  | *Hemicriconemoides wessoni* | KJ934166 |  |  |  |
|  | *Hemicycliophora_cardamomi* |  | MW001621 |  |  |
|  |  |  |  | MW000897 |  |
|  | *Hemicycliophora conida* | AJ966471 |  |  |  |
|  |  |  | MN628433 |  |  |
|  |  |  |  | MG019904 |  |
|  | *Hemicycliophora subbotini* | MG701279 |  |  |  |
|  | *Lobocriconema incrassatum* | MF095018 |  |  | These sequences belong to a single species and morphological data of these nematode populations need to be reviewed |
|  |  | MF095014 |  |  |  |
|  |  |  |  | KU236506 |  |
|  | *Lobocriconema thornei* | MF094994 |  |  |  |
|  |  | MF094928 |  |  |  |
|  |  |  |  | KU236625 |  |
|  |  |  |  | KU236532 |  |
|  |  |  |  | KU236522 |  |
|  | *Lobocriconema iranense* | MK546401 |  |  |  |
|  |  |  | MK546404 |  |  |
|  |  |  | MK546406 |  |  |
|  |  |  | MK546405 |  |  |
|  |  |  | MK546408 |  |  |
|  |  |  | MK546407 |  |  |
|  |  |  |  | MK546389 |  |
|  |  |  |  | MK546390 |  |
|  |  |  |  | MK546391 |  |
|  | *Lobocriconema nokandense* | MK546402 |  |  | These sequences represent *L. iranense* |
|  |  |  | MK546410 |  |  |
|  |  |  | MK546411 |  |  |
|  |  |  | MK546409 |  |  |
|  |  |  |  | MK546394 |  |
|  | *Lobocriconema warrenense* |  |  | KU236545 |  |
|  |  |  |  | KU236547 |  |
|  |  |  |  | KU236546 |  |
|  | *Mesocriconema antipolitanum* |  | MN888462 |  |  |
|  |  |  | MN888461 |  |  |
|  |  |  | MN888460 |  |  |
|  | *Mesocriconema curvatum* | MF094891 |  |  |  |
|  |  |  | MN720094 |  |  |
|  | *Mesocriconema discus* | MF094892 |  |  | This sequence represents *M. xenoplax* |
|  | *Mesocriconema ericaceum* | KX290604 |  |  | These sequences represent *Mesocriconema xenoplax* |
|  |  | KX290603 |  |  |  |
|  | *Mesocriconema inaratum* | MF094903 |  |  | This sequence represents *Mesocriconema xenoplax* |
|  | *Mesocriconema nebraskense* | KY574844 |  |  | These sequences represent *Mesocriconema xenoplax* |
|  |  | KY574845 |  |  |  |
|  |  |  | MN720085 |  |  |
|  |  |  | MH013430 |  |  |
|  |  |  | MN720087 |  |  |
|  | *Mesocriconema onoense* | MZ361704 |  |  |  |
|  |  | MF094909 |  |  |  |
|  |  |  | MZ220549 |  |  |
|  | *Mesocriconema ornatum* | MF094893 |  |  |  |
|  |  |  | MW938536 |  |  |
|  |  |  | MW938535 |  |  |
|  |  |  | MW938534 |  |  |
|  | *Mesocriconema rusticum* | MF094965 |  |  |  |
|  |  |  |  | KJ787855 |  |
|  | *Mesocriconema sphaerocephalum* | KJ934182 |  |  | This sequence was misidentified |
|  |  | MF094912 |  |  |  |
|  |  | MF094921 |  |  |  |
|  |  | MW254992 |  |  |  |
|  |  | MW254991 |  |  |  |
|  |  |  | MZ262318 |  | This sequence represents *Criconema mutabile* |
|  |  |  | AB933466 |  |  |
|  |  |  | MK026628 |  |  |
|  |  |  |  | MN711169 |  |
|  |  |  |  | MN711168 |  |
|  | *Mesocriconema xenoplax* | KJ934180 |  |  |  |
|  |  | AY284625 |  |  |  |
|  |  | MF094896 |  |  |  |
|  |  | KJ934176 |  |  |  |
|  |  |  | KC538862 |  | These sequences represent *M. onoense* |
|  |  |  | FN433873 |  |  |
|  |  |  | MZ262315 |  |  |
|  |  |  | MN056431 |  |  |
|  |  |  | FN433853 |  |  |
|  |  |  | FN433861 |  |  |
|  |  |  | FN433859 |  |  |
|  |  |  | FN600558 |  |  |
|  |  |  | FN433869 |  |  |
|  |  |  | FN433856 |  |  |
|  |  |  | MN783683 |  |  |
|  |  |  | FN433867 |  |  |
|  | *Neobakernema variabile* |  |  | MF683242 |  |
|  | *Neolobocriconema serratum* |  |  | MH668969 |  |
|  |  |  |  | KU236634 |  |
|  | *Nothocriconemoides hangzhouensis* |  | MW938522 |  |  |
|  |  |  | MW938523 |  |  |
|  | *Ogma cobbi* | EU669918 |  |  |  |
|  | *Ogma decalineatus* | MF094952 |  |  |  |
|  |  |  | MW938539 |  |  |
|  |  |  | MW938537 |  |  |
|  |  |  |  | MN711263 |  |
|  |  |  |  | MN711262 |  |
|  | *Ogma hechuanensis* |  | MW938515 |  |  |
|  |  |  | MW938513 |  |  |
|  | *Ogma menzeli* | EU669919 |  |  | This sequence represents *Crossonema fimbriatum* |
|  | *Ogma murrayi* | MF094930 |  |  |  |
|  |  |  |  | MN711282 |  |
|  |  |  |  | MN711281 |  |
|  |  |  |  | MN711279 |  |
|  |  |  |  | MN711278 |  |
|  | *Ogma octangularis* | MW938284 |  |  | Other taxonomical data are needed to confirm the status of these sequences |
|  |  | MF094954 |  |  |  |
|  |  | MF094956 |  |  |  |
|  | *Ogma seymouri* | MF094933 |  |  |  |
|  |  | KX344498 |  |  |  |
|  |  |  |  | MN711328 |  |
|  |  |  |  | MN711327 |  |
|  |  |  |  | KX290586 |  |
|  |  |  |  | KX290580 |  |
|  | *Ogma tenuicaudatus* |  |  | MN711341 |  |
